# Supplementary material for: Retirement ages of senior UK doctors: national surveys of the medical graduates of 1974 and 1977
Source: BMJ Open. 2018 Jun 27;8(6):e022475. doi: 10.1136/bmjopen-2018-022475 (PMC6042587; doi:10.1136/bmjopen-2018-022475)
Supplement: Supplementary file 1 [file bmjopen-2018-022475supp001.pdf]

Supplementary Table 1: Employment status at time of survey of the graduates of 1974, by gender and specialty

		Employment status at time of survey									
		Full-time in medicine		Part-time in medicine		'Retired and returned'		Retired		Total	
		N	%	N	%	N	%	N	%	N	%
General practice	Men	40	7.9	62	12.3	97	19.2	306	60.6	505	100.0
	Women	9	4.3	20	9.5	26	12.4	155	73.8	210	100.0
Hospital medicine	Men	46	25.8	19	10.7	59	33.1	54	30.3	178	100.0
	Women	8	9.8	4	4.9	16	19.5	54	65.9	82	100.0
Surgery	Men	40	21.5	11	5.9	76	40.9	59	31.7	186	100.0
	Women	4	15.4	1	3.8	5	19.2	16	61.5	26	100.0
Anaesthesia	Men	9	14.5	5	8.1	18	29.0	30	48.4	62	100.0
	Women	2	11.1	1	5.6	2	11.1	13	72.2	18	100.0
Psychiatry	Men	11	22.9	2	4.2	19	39.6	16	33.3	48	100.0
	Women	1	3.6	2	7.1	13	46.4	12	42.9	28	100.0
Pathology	Men	3	7.3	3	7.3	12	29.3	23	56.1	41	100.0
	Women	1	6.3	1	6.3	5	31.3	9	56.3	16	100.0
Radiology	Men	6	20.7	1	3.4	9	31.0	13	44.8	29	100.0
	Women	1	7.7	0	0.0	4	30.8	8	61.5	13	100.0
Total	Men	155	14.8	103	9.8	290	27.6	501	47.8	1049	100.0
	Women	26	6.6	29	7.4	71	18.1	267	67.9	393	100.0

Supplementary Table 2: Employment status at time of survey of the graduates of 1977, by gender and specialty

		Employment status in 2014									
		Full-time in medicine		Part-time in medicine		'Retired and returned'		Retired		Total	
		N	%	N	%	N	%	N	%	N	%
General practice	Men	97	15.5	87	13.9	184	29.5	256	41.0	624	100.0
	Women	24	7.1	59	17.5	62	18.4	192	57.0	337	100.0
Hospital medicine	Men	91	42.5	18	8.4	66	30.8	39	18.2	214	100.0
	Women	22	20.4	13	12.0	25	23.1	48	44.4	108	100.0
Surgery	Men	111	45.1	19	7.7	70	28.5	46	18.7	246	100.0
	Women	13	28.3	7	15.2	14	30.4	12	26.1	46	100.0
Anaesthesia	Men	23	23.0	6	6.0	31	31.0	40	40.0	100	100.0
	Women	7	20.6	1	2.9	7	20.6	19	55.9	34	100.0
Psychiatry	Men	9	16.7	3	5.6	21	38.9	21	38.9	54	100.0
	Women	8	12.9	7	11.3	25	40.3	22	35.5	62	100.0
Pathology	Men	21	32.8	1	1.6	22	34.4	20	31.3	64	100.0
	Women	3	8.1	4	10.8	6	16.2	24	64.9	37	100.0
Radiology	Men	16	31.4	3	5.9	20	39.2	12	23.5	51	100.0
	Women	5	31.3	4	25.0	3	18.8	4	25.0	16	100.0
Total	Men	368	27.2	137	10.1	414	30.6	434	32.1	1353	100.0
	Women	82	12.8	95	14.8	142	22.2	321	50.2	640	100.0
