# Supplementary material for: Retirement ages of senior UK doctors: national surveys of the medical graduates of 1974 and 1977
Source: BMJ Open. 2018 Jun 27;8(6):e022475. doi: 10.1136/bmjopen-2018-022475 (PMC6042587; doi:10.1136/bmjopen-2018-022475)
Supplement: Author's manuscript [file bmjopen-2018-022475.draft_revisions.pdf]

BMJ Open is committed to open peer review. As part of this commitment we make the peer review history of every article we publish publicly available.

When an article is published we post the peer reviewers' comments and the authors' responses online. We also post the versions of the paper that were used during peer review. These are the versions that the peer review comments apply to.

The versions of the paper that follow are the versions that were submitted during the peer review process. They are not the versions of record or the final published versions. They should not be cited or distributed as the published version of this manuscript.

BMJ Open is an open access journal and the full, final, typeset and author-corrected version of record of the manuscript is available on our site with no access controls, subscription charges or pay-per-view fees (http://bmjopen.bmj.com).

If you have any questions on BMJ Open's open peer review process please email info.bmjopen@bmj.com

BMJ Open

Retirement ages of senior UK doctors: national surveys of the medical graduates of 1974 and 1977

Journal:	*BMJ Open*
Manuscript ID	bmjopen-2018-022475
Article Type:	Research
Date Submitted by the Author:	19-Feb-2018

Complete List of Authors:	Smith, Fay; University of Oxford, Nuffield Department of Population Health<br>Goldacre, Michael; University of Oxford, Nuffield Department of Population Health<br>Lambert, Trevor; University of Oxford, Nuffield Department of Population Health

Keywords:	MEDICAL EDUCATION & TRAINING, Physicians, workforce, retirement

SCHOLARONE™ Manuscripts

For peer review only

Retirement ages of senior UK doctors: national surveys of the medical graduates of 1974 and 1977

Short title: Retirement ages of senior UK doctors

Fay Smith PhD[1], Michael J Goldacre FFPH[1] , Trevor W Lambert MSc[1]

[1]UK Medical Careers Research Group, Unit of Health-Care Epidemiology, Nuffield Department of Population Health, University of Oxford, Old Road Campus, Oxford OX3 7LF

Corresponding author: Trevor Lambert (trevor.lambert@dph.ox.ac.uk; Telephone: 01865 289389)

Word count: 2346 words.

For peer review only

ABSTRACT

Objective: To report on retirement ages of two cohorts of senior doctors in the latter stages of their careers.

Design: Questionnaires sent in 2014 to all medical graduates of 1974 and 1977.

Setting: UK.

Participants: 3695 UK medical graduates.

Main outcome measures: Retirement status by age at the time of the survey, and age at retirement if retired. Planned and contractual retirement ages, and retirement plans if not retired.

Results: Of contactable doctors, 85% responded. 43.7% of all responding doctors had fully retired, 25.9% had 'retired and returned' for some medical work, 18.3% had not retired and were working full-time in medicine, 10.7% had not retired and were working part-time in medicine, and 1.4% were either doing non-medical work or did not give details of their employment status. The average actual retirement age (including those who had retired but subsequently returned) was 59.6 years (men 59.9, women 58.9). Psychiatrists (58.3) and GPs (59.5) retired at a slightly younger age than radiologists (60.4), surgeons (60.1) and hospital specialists (60.0). More GPs (54%) than surgeons (26%) or hospital medical specialists (34%) were fully retired, and there were substantial variations in retirement rates in other specialties. Sixty-three percent of women GPs were fully retired. Retired doctors had left, on average, 2.5 years before their contractual retirement age.

Conclusions: Gender and specialty differences in retirement ages were apparent and are worthy of qualitative study to establish underlying reasons in those specialties where early retirement is more common. There is a general societal expectation that people will retire at increasingly elderly ages; but the doctors in this national study retired relatively young.

[267 words]

For peer review only - http://bmjopen.bmj.com/site/about/guidelines.xhtml

Strengths and limitations of this study

- This is large scale study of graduates from all UK medical schools.
- The study has a very high response rate (85%) for a questionnaire study with voluntary participation.
- However there is a possibility of non-responder bias.

For peer review only

For peer review only - http://bmjopen.bmj.com/site/about/guidelines.xhtml

INTRODUCTION

In the United Kingdom (UK), as in many other countries, as average life expectancy increases, retirement age and pensionable age will have to increase too. The World Economic Forum has reported that a baby born in 2017 can expect to live to over 100, and more immediately, retirement systems that were designed to support a retirement of 10-15 years will have to change to prepare for a "seismic shift" as without substantially increasing retirement ages, individuals would eventually be spending more time retired than working.[1] Already changes are happening in the UK: for example the state pension age is rising to 66 in 2020 and to 67 by 2028.[2]

Against this background, and the trend to work longer to ensure financial security in old age, there are data which suggest that older doctors, both in the UK and in other countries, far from working longer, are in fact retiring early or reducing their working hours in increasing numbers in recent years.[3 4] A recent survey of consultant physicians in the UK found that their average intended age of retirement was 62 years, and that 72% of these doctors did not intend to work beyond retirement age.[5] Early retirement of doctors has the major consequence for health-care of early loss of doctors from the medical workforce.

In 2014, 20% of UK consultants and 21% of General Practitioners (GPs) were aged 55 or over.[6] The median retirement age for GPs in the UK was 58 for women and 60 for men, between the years 2008 and 2011.[7]

The aim of this paper is to report on the retirement status and ages, and retirement intentions, of two cohorts of senior doctors who graduated in 1974 and 1977 whom we surveyed in 2014 when they had median ages of 64 and 61 years old respectively. We compared the replies of men and women, of those in the two cohorts, and of those working in different career specialties.

METHODS

The UK Medical Careers Research Group surveyed the UK medical graduates of 1974 and 1977. This was our last planned survey in career-long studies of the professional choices and work trajectories of these cohorts.[8-14] We sent postal and web-based questionnaires to these senior doctors in 2014. Up to four reminders were sent to non-respondents. Further details of the methodology are available elsewhere.[15]

For peer review only

For peer review only - http://bmjopen.bmj.com/site/about/guidelines.xhtml

The surveys sent to both cohorts were identical and comprised structured, 'closed' questions and statements. Doctors were asked to indicate their current employment status, choosing from seven categories (working in medicine full-time or part-time, working outside medicine full-time or part-time, retired, 'retired and returned' for some medical work, or other). Doctors were then asked to respond to questions about their retirement age (if they had retired), or their retirement plans (if still working; see Box 1).

Box 1: Questions and statements presented to respondents

Asked of	Focus	Question/Statement
Retirees and doctors who had 'retired and returned'	Retirement age	1. How old were you when you retired?<br>2. What was the normal contractual retirement age for your job?
Doctors still working in medicine (full or part-time)	Retirement plans	3. At what age do you plan to retire?<br>4. What is the fixed retirement age for your post, if you know it?<br>5. Do you plan to reduce your time commitments to your work before retiring? [Yes, I have already done so; Yes, in the future; No; Don't know]

We allocated a career specialty to each respondent using their recorded job history as reported to us in successive surveys and additional information about their specialist registration with the GMC as reported to us by the doctors in these surveys. For a small number of respondents we were unable to allocate a single career specialty, either because we did not have sufficient data about the doctor's career, or because the doctor had worked in different specialties during their career. The career specialty allocation allowed us to analyse the responses and employment status of doctors in different specialties. Respondents were then grouped for analysis into seven groups: general practice, hospital medicine (comprising the hospital physician specialties and paediatrics), surgery (comprising the surgical specialties, emergency medicine, obstetrics and gynaecology, and clinical oncology), anaesthesia, psychiatry, pathology, and radiology.

The replies were analysed using χ^2 tests and Mann-Whitney U tests to explore differences between male and female doctors, between cohorts and between doctors working in different specialties.

During ethical approval we obtained permission to approach doctors for participation and to take their response as an indicator of informed consent to participate in the studies.

For peer review only - http://bmjopen.bmj.com/site/about/guidelines.xhtml

RESULTS

Response rates

We were able to contact 4369 of the original 5482 UK medical graduates from 1974 and 1977 (Table 1). The aggregated response rate from both cohorts was 84.6% (3695/4369). To maximise response, at the end of the survey period non-responders were given the option of completing a shortened questionnaire: 98 did so, and because of the curtailed information obtained from them, they could not be included in some of the analysis below.

Table 1: UK doctors who graduated in 1974 and 1977: response to survey, and career status of respondents

TARGET GROUPING	Year of graduation 1974	1977	Total
GRADUATION COHORT	2347 (100%)	3135 (100%)	5482 (100%)
Known to be deceased	*100 (4.3%)*	*110 (3.5%)*	*210 (3.8%)*
Declined to participate	*20 (0.9%)*	*50 (1.6%)*	*70 (1.3%)*
Uncontactable	*415 (17.7%)*	*418 (13.3%)*	*833 (15.2%)*
Contacted	*1812 (77.2%)*	*2557 (81.6%)*	*4369 (79.7%)*
CONTACTABLE DOCTORS	1812 (100%)	2557 (100%)	4369 (100%)
Did not respond	*267 (14.7%)*	*407 (15.9%)*	*674 (15.4%)*
Responded in brief	*47 (2.6%)*	*51 (2.0%)*	*98 (2.2%)*
Responded in full	*1498 (82.7%)*	*2099 (82.1%)*	*3597 (82.3%)*
RESPONDENTS	1545 (100%)	2150 (100%)	3695 (100%)
Working full-time in medicine	*195 (12.6%)*	*483 (22.5%)*	*678 (18.3%)*
Working part-time in medicine	*145 (9.4%)*	*252 (11.7%)*	*397 (10.7%)*
Working full-time outside medicine	*6 (0.4%)*	*10 (0.5%)*	*16 (0.4%)*
Working part-time outside medicine	*7 (0.5%)*	*9 (0.4%)*	*16 (0.4%)*
Retired, not now working in medicine	*804 (52.0%)*	*810 (37.7%)*	*1614 (43.7%)*
Retired, and 'returned' for some medical work	*380 (24.6%)*	*577 (26.8%)*	*957 (25.9%)*
Other / no reply	*8 (0.5%)*	*9 (0.4%)*	*17 (0.5%)*

Employment status in 2014

In 2014, 43.7% of all responding doctors had retired, 25.9% had 'retired and returned' for some medical work, 18.3% were working full-time in medicine, 10.7% were working part-time in medicine, and 1.3% were either doing non-medical work or did not give details of their employment status (Table 1). Having graduated three years earlier than the 1977 graduates, more doctors from the 1974 cohort (52.0%) had retired than from the 1977 cohort (37.7%).

In further analysis, we excluded doctors whose career specialty (see Method) was unknown, or who were working outside medicine, or who did not declare their employment status, and analysed the employment status in 2014 of the 3435 respondents remaining, by gender and specialty grouping (Table 2).

For peer review only - http://bmjopen.bmj.com/site/about/guidelines.xhtml

Table 2: Career specialty, and employment status at time of survey, of graduates of 1974 and 1977

	Employment status				
	Full-time in medicine	Part-time in medicine	'Retired and returned'	Retired	Total N (100%)
	%	%	%	%	%
Total	18.4	10.6	26.7	44.3	3435
General practice	10.1	13.6	22.0	54.2	1676
Hospital medicine	28.7	9.3	28.5	33.5	582
Surgery	33.3	7.5	32.7	26.4	504
Anaesthesia	19.2	6.1	27.1	47.7	214
Psychiatry	15.1	7.3	40.6	37.0	192
Pathology	17.7	5.7	28.5	48.1	158
Radiology	25.7	7.3	33.0	33.9	109
Women	10.5	12.0	20.6	56.9	1033
General practice	6.0	14.4	16.1	63.4	547
Hospital medicine	15.8	8.9	21.6	53.7	190
Surgery	23.6	11.1	26.4	38.9	72
Anaesthesia	17.3	3.8	17.3	61.5	52
Psychiatry	10.0	10.0	42.2	37.8	90
Pathology	7.5	9.4	20.8	62.3	53
Radiology	20.7	13.8	24.1	41.4	29
Men	21.8	10.0	29.3	38.9	2402
General practice	12.1	13.2	24.9	49.8	1129
Hospital medicine	34.9	9.4	31.9	23.7	392
Surgery	35.0	6.9	33.8	24.3	432
Anaesthesia	19.8	6.8	30.2	43.2	162
Psychiatry	19.6	4.9	39.2	36.3	102
Pathology	22.9	3.8	32.4	41.0	105
Radiology	27.5	5.0	36.3	31.3	80

Percentages are row percentages.
Excludes 32 respondents working in non-medical jobs and 228 whose employment status and/or career specialty was unknown.
See Method for explanation of career specialty grouping.
For detailed specialty breakdown in each cohort, with numbers and percentages, see Supplementary Tables 1 and 2.

Taking men and women together, employment status and career specialty were strongly related (χ^2_{18}=324.1, p<.001): the most notable specialty differences were that more general practitioners (54%), and fewer surgeons (26%) and hospital medical specialists (34%), were fully retired compared to the overall average; and fewer general practitioners (10%) and more surgeons (33%) and hospital medical specialists (29%) were working full-time compared to the overall average (based on analysis of adjusted residuals).

For peer review only

Comparing specialties separately for men and women (Table 2), the specialty differences in employment status for men were significant (χ^2_{18}=2241.7, p<.001), and in the same specialties as the total. For women, the specialty differences in employment status were also significant (χ^2_{18}=84.3, p<.001); but the percentage of women hospital medical specialists who were retired (54%) was much closer to the percentage of women general practitioners (63%) who were retired, than was the case for the men (24% vs 50%).

Supplementary tables 1 and 2 show results for the 1974 and 1977 cohorts separately. In many cases the numbers in individual specialties are too small for meaningful statistical comparisons to be made.

Age at retirement

Figure 1 shows the percentages of men and women respondents in each cohort who had not retired and who were working in medicine at each year of age. (The figure treats 'retired and returned' as not working in medicine for this purpose). Log rank tests showed that the male-female difference was significant in the 1974 cohort (χ^2_1=93.9, p<.001) and in the 1977 cohort (χ^2_1=78.8, p<.001): at any age the percentage of women who were retired was larger than that of men. Within gender, the two cohorts followed similar patterns of retirement by age (women χ^2_1=0.3, p=.57; men χ^2_1=0.7, p=.39).

Figures 2 and 3 show the data for men and women separately, subdivided by career specialty, based on the seven groups previously defined (see Method). The cohorts have been combined but the gender separation has been retained, in view of the results from Figure 1. Specialty differences were more marked for men (χ^2_1=130.7, Figure 2) than for women (χ^2_1=25.1, Figure 3), both p<.001. Within each specialty, the gender difference was significant with p<.001 for general practice and hospital medicine, and was borderline significant for surgery, anaesthesia, and pathology (in each case with higher percentages of women than men having retired): there was no gender difference for psychiatry or radiology (both p>.05).

Retired doctors: actual and contractual retirement ages

We asked doctors their actual retirement age (if they had retired) and their contracted retirement age. The average actual retirement age (including those who had retired but subsequently returned) was 59.6 years (59.9 men, 58.9 women; 60.4 1974 cohort, 58.9 1977 cohort). Retired doctors left, on average, 2.5 years before their contractually specified retirement age. Psychiatrists (58.3) and GPs (59.5) retired at a slightly younger age than

For peer review only - http://bmjopen.bmj.com/site/about/guidelines.xhtml

radiologists (60.4), surgeons (60.1) and hospital specialists (60.0) (χ^2_6=67.8, p<.001; Kruskal Wallis test).

Doctors who had not retired and who were still working in medicine: planned and contractual retirement ages

The average planned retirement age of doctors still working was 65.7 years (66.0 men, 64.7 women; 67.4 1974 cohort, 64.9 1977 cohort; 65.9 full-time, 65.3 part-time). Part-time workers planned to retire 2.2 years before their contractually specified retirement age, compared with 0.3 years before for full-time workers. The difference between planned and contractual retirement ages was the same for men and women doctors (0.9 years). Psychiatrists still working planned to retire at an older age (67.4) than doctors in other specialties: anaesthetists (64.5), pathologists (64.9), GPs (65.2) and radiologists (65.2) (χ^2_6=26.0, p<.001; Kruskal Wallis test).

Doctors who had not retired and who were still working: retirement plans

When asked 'Do you plan to reduce your time commitments to your work before retiring?' (38.2%) of doctors who were still working answered 'Yes, I have already done so', 29.2% answered 'Yes, in the future', 22.2% answered 'No', and 10.4% answered 'Don't know'. More men (32.2%) than women (19.5%) answered 'Yes, in the future' (χ^2_4=21.5, p<.001). There were significant differences between specialties (χ^2_{18}=69.9, p<.001). More GPs (53.1%) answered 'Yes, I have already done so' than surgeons (29.7%) and doctors in hospital medicine (29.3%).

DISCUSSION

Main findings

Given that these doctors were predominantly only in their early sixties, the level of retirement was surprisingly high given societal changes in life expectancy and retirement planning. 'Retiring and returning', often to a less demanding or less time-committed post, and substantial levels of part-time working among the remainder, meant that there remained only a small minority of these highly experienced doctors working full-time in medicine.

Specialty differences in retirement levels at the time of the survey were seen for both men and women. They were more pronounced for men, with male hospital doctors having low levels of retirement compared to other men, or women. This may reflect gender roles and

For peer review only - http://bmjopen.bmj.com/site/about/guidelines.xhtml

expectation in this older generation, factors which may not apply to the same extent to younger generations when they reach the equivalent stages in their careers.

Retirement profiles by age showed similar results and, additionally, in general practice, the hospital medical specialties, surgery, anaesthesia, and pathology, the proportion of women who had retired increased more with increasing age than was the case for men.

Those who had retired had on average done so 2.5 years before their contractual retirement age and changes in the work environment and in personal circumstances were often contributory factors.

GPs who were still working were more inclined to have planned work reductions than their hospital colleagues.

Strengths and limitations

This is large scale study of graduates from all UK medical schools. The study has a very high response rate (85%) for a questionnaire study with voluntary participation. However there is a possibility of non-responder bias.

In 2017, in the UK 47% of the registered UK medical workforce were women: of doctors aged under 30 years (13% of the whole workforce), 62% were women.[16] By contrast, in our study women constituted only 30% of the 1974/1977 cohorts. We cannot judge whether younger cohorts, with higher percentages of women, will eventually show similar profiles of retirement to the subjects in our study.

Comparison with existing literature

The doctors we surveyed retired, on average, aged 60 – two years earlier than the average intended retirement age given by consultants in a UK census conducted in 2014-5.[5] The UK census however comprises consultants who are older than the 1974 and 1977 cohorts we studied. On 1 February 2018 Pulse Magazine reported that analysis of figures received for NHS Business Services Authority under a Freedom of Information request showed that 62% of GPs claiming their pension for the first time in 2016/17 were under 60 years old, compared with 33% in 2011/12. A review of studies which reported both intended and actual retirement ages found the two ages to be similar.[17] As mentioned in the Introduction, there is a general societal trend towards later retirement (which contrasts with our finding of doctors' earlier retirement), with UK women in particular working longer as the state pension

For peer review only

For peer review only - http://bmjopen.bmj.com/site/about/guidelines.xhtml

age increases.[18-20] An earlier analysis of these cohorts, reporting a median age of 64 for those who retired in 2014, found that 27% had retired earlier than they had originally intended; and, of those who had retired, their main reasons for retirement were 'increased time for leisure/other interests' and 'pressure of work'.[21] A systematic review of physician retirement identified burnout and excessive workload as the main reasons for early retirement.[17]

We found that more GPs (54%) than doctors in other specialties were retired, and 63% of women GPs were retired. Factors influencing GPs' intentions to leave general practice include workload intensity, bureaucracy, appraisal and revalidation.[22-24] Research in Australia of doctors aged over 55 found that psychiatrists and GPs were less likely than doctors in other specialties to say that they intended to retire,[25] and research in Canada found no difference in retirement age between specialties.[26] In the UK, GPs are reported to have had higher levels of burnout than doctors in other specialties.[27]

Implications / conclusions

Our findings demonstrate the scale of loss of doctors from the workforce as a result of retirement in the early 60s. Premature loss of experienced doctors in late career has an adverse impact on workforce capacity and it needs to be better understood. Nonetheless, it is difficult to untangle the motivations of doctors as they consider and plan the reality and timing of their retirement. Every retirement, like every medical career, will be individual and will be the outcome of a number of considerations related to work, home life and leisure. In these cohorts, women are retiring younger than men; retirement ages were on average two to three years younger than contracted ages; and there was substantial variation by specialty. There is scope for further study of underlying reasons for early retirement, and in particular to address the question of whether those attracted to some specialties are more likely than others to have characteristics which predispose them to early retirement, or whether there are inherent issues in some specialties which render early retirement more attractive. If the latter, policy amendments to ameliorate these issues may reduce workforce losses consequent to early retirement.

[2346 words]

For peer review only - http://bmjopen.bmj.com/site/about/guidelines.xhtml

Conflict of interests: All authors have completed the Unified Competing Interest form at www.icmje.org/coi_disclosure.pdf (available on request from the corresponding author) and all authors want to declare: (1) financial support for the submitted work from the policy research programme, Department of Health. All authors also declare: (2) no financial relationships with commercial entities that might have an interest in the submitted work; (3) no spouses, partners, or children with relationships with commercial entities that might have an interest in the submitted work; (4) no non-financial interests that may be relevant to the submitted work.

Funding: The report is based on independent research commissioned and funded by the NIHR Policy Research Programme (project number 016/0118). The views expressed in the publication are those of the author(s) and not necessarily those of the NHS, the NIHR, the Department of Health, its arm's length bodies or other government departments.

Data sharing statement: It may be possible for the authors to make tabulated data, produced in the course of this work but not included in the paper, available to interested readers on request.

Ethical approval: National Research Ethics Service, following referral to the Brighton and Mid-Sussex Research Ethics Committee in its role as a multi-centre research ethics committee (ref 04/Q1907/48 amendment Am02 March 2015).

Guarantor: All authors are guarantors.

Contributorship: TL and MJG designed and conducted the surveys. FS performed the analysis and wrote the first drafts of the paper. All authors contributed to further drafts and all approved the final version.

Acknowledgements: We thank Shelly Lachish for early work on the paper and Janet Justice and Alison Stockford for data entry. We are very grateful to all the doctors who participated in the surveys.

MeSH terms: Physicians; workforce; medical; retirement.

What this paper adds

For peer review only - http://bmjopen.bmj.com/site/about/guidelines.xhtml

What is already known

- There is a societal trend for retirement ages and pensionable ages to increase with increased life expectancy
- However, some studies have suggested that doctors are retiring early in increasing numbers, or are reducing their work commitments in the later stages of their careers

What this study adds

- Many doctors in our study had retired completely by their early 60s, or had 'retired and returned' to a less demanding or time-committed post
- However, there was substantial variation by specialty and by gender

For peer review only

For peer review only - http://bmjopen.bmj.com/site/about/guidelines.xhtml

REFERENCES

1. World Economic Forum. We'll Live to 100 – How Can We Afford It? Geneva: World Economic Forum, 2017.

2. Department for Work and Pensions. State Pension age timetable. London: Department for Work and Pensions, 2013.

3. Royal College of Physicians. Census of consultant physicians and higher specialty trainees in the UK 2016-17. London: Royal Colleges of Physicians, 2017.

4. Joyce CM, Wang WC, McDonald HM. Retirement patterns of Australian doctors aged 65 years and older. Aust. Health Rev. 2015;**39**(5):582-87 doi: 10.1071/AH14176 [published Online First: 22 June 2015].

5. Royal College of Physicians. Census of consultant physicians and higher specialty trainees in the UK 2014-15. London: Royal Colleges of Physicians, 2016.

6. British Medical Association. 2014 UK Medical Workforce Briefing. London: British Medical Association, 2014.

7. Centre for Workforce Intelligence. In-depth review of the general practitioner workforce. London: Centre for Workforce Intelligence, 2014.

8. Parkhouse J, McLaughlin C. Career preferences of doctors graduating in 1974. Br. Med. J. 1976;**2**(6036):630-2 1976/09/11].

9. Parkhouse J, Palmer MK, Hambleton BA. Career preferences of doctors qualifying in the United Kingdom in 1977. Health Trends 1979;**11**(2):35-7.

10. Parkhouse J, Campbell MG, Parkhouse HF. Careers of doctors qualifying in Britain in 1974: a report on their employment status in 1979. Br. Med. J. (Clin. Res. Ed). 1982;**285**(6340):522-6 1982/08/14].

11. Davidson JM, Lambert TW, Goldacre MJ. Career pathways and destinations 18 years on among doctors who qualified in the United Kingdom in 1977: Postal questionnaire survey. Br. Med. J. 1998;**317**(7170):1425-28.

12. Taylor K, Lambert T, Goldacre M. Future career plans of a cohort of senior doctors working in the National Health Service. J. R. Soc. Med. 2008;**101**(4):182-90.

13. Taylor K, Lambert T, Goldacre M. Career destinations, job satisfaction and views of the UK medical qualifiers of 1977. J. R. Soc. Med. 2008;**101**(4):191-200.

14. Goldacre MJ, Laxton L, Lambert TW. Medical graduates' early career choices of specialty and their eventual specialty destinations: UK prospective cohort studies. BMJ 2010;**341**(7764):139 doi: 10.1136/bmj.c3199 [published Online First: 06 July 2010].

For peer review only - http://bmjopen.bmj.com/site/about/guidelines.xhtml

15. Goldacre M, Lambert T. Participation in medicine by graduates of medical schools in the United Kingdom up to 25 years post graduation: National cohort surveys. Acad. Med. 2013;**88**(5):699-709 doi: 10.1097/ACM.0b013e31828b364f [published Online First: 01 May 2013].

16. General Medical Council. The state of medical education and practice in the UK report: 2017. London: GMC, 2017.

17. Silver MP, Hamilton AD, Biswas A, et al. A systematic review of physician retirement planning. Human Resources for Health 2016;**14**(1):67 doi: 10.1186/s12960-016-0166-z [published Online First: 15 November 2016].

18. Emmerson C, Heald K, Hood A. The Changing Face of Retirement: Future Patterns of Work, Health, Care and Income among the Older Population. London: Institute for Fiscal Studies, 2014.

19. Houses of Parliament: parliamentary office of science and technology. An Ageing Workforce. London: Houses of Parliament, 2011.

20. Demou E, Bhaskar A, Xu T, et al. Health, lifestyle and employment beyond state-pension age. BMC Public Health 2017;**17**(1) doi: 10.1186/s12889-017-4957-5 [published Online First: 20 December 2017].

21. Smith F, Lachish S, Goldacre MJ, et al. Factors influencing the decisions of senior UK doctors to retire or remain in medicine: national surveys of the UK-trained medical graduates of 1974 and 1977. BMJ open 2017;**7**(9) doi: 10.1136/bmjopen-2017-017650 [published Online First: 1 November 2017].

22. Dale J, Potter R, Owen K, et al. The general practitioner workforce crisis in England: A qualitative study of how appraisal and revalidation are contributing to intentions to leave practice. BMC Family Practice 2016;**17**(1) doi: 10.1186/s12875-016-0489-9 [published Online First: 20 July 2016].

23. Dale J, Potter R, Owen K, et al. Retaining the general practitioner workforce in England: What matters to GPs? A cross-sectional study. BMC Family Practice 2015;**16**(1) doi: 10.1186/s12875-015-0363-1 [published Online First: 16 October 2015].

24. Sansom A, Calitri R, Carter M, et al. Understanding quit decisions in primary care: A qualitative study of older GPs. BMJ Open 2016;**6**(2):e010592 doi: 10.1136/bmjopen-2015-010592 [published Online First: 19 February 2016].

25. Wijeratne C, Earl JK, Peisah C, et al. Professional and psychosocial factors affecting the intention to retire of Australian medical practitioners. Med. J. Aust. 2017;**206**(5):209-14 doi: 10.5694/mja16.00883 [published Online First: 20 March 2017].

For peer review only - http://bmjopen.bmj.com/site/about/guidelines.xhtml

26. Hedden L, Lavergne MR, McGrail KM, et al. Patterns of physician retirement and pre-retirement activity: A population-based cohort study. CMAJ 2017;**189**(49):E1517-E23 doi: 10.1503/cmaj.170231 [published Online First: 11 December 2017].
27. Halliday L, Walker A, Vig S, et al. Grit and burnout in UK doctors: A cross-sectional study across specialties and stages of training. Postgrad. Med. J. 2017;**93**(1101):389-94 doi: 10.1136/postgradmedj-2015-133919 [published Online First: 18 November 2016].

For peer review only

For peer review only - http://bmjopen.bmj.com/site/about/guidelines.xhtml

FIGURE CAPTIONS

Figure 1: Percentage of 1974 and 1977 graduates who had not retired and who were working in medicine, by age: Kaplan-Meier estimates

Short legend: Doctors working in medicine, by age

Figure 2: Male 1974 and 1977 graduates still working in medicine, percentages by age: Kaplan-Meier estimates

Short legend: Male doctors working in medicine, by specialty

Figure 3: Female 1974 and 1977 graduates still working in medicine, percentages by age: Kaplan-Meier estimates

Short legend: Female doctors working in medicine, by specialty

For peer review only

For peer review only - http://bmjopen.bmj.com/site/about/guidelines.xhtml

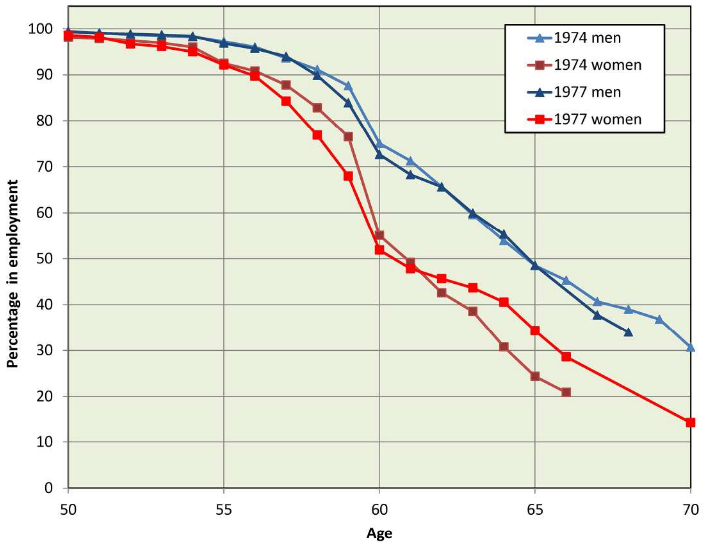

Figure 1: Percentage of 1974 and 1977 graduates who had not retired and who were working in medicine, by age: Kaplan-Meier estimates

209x148mm (300 x 300 DPI)

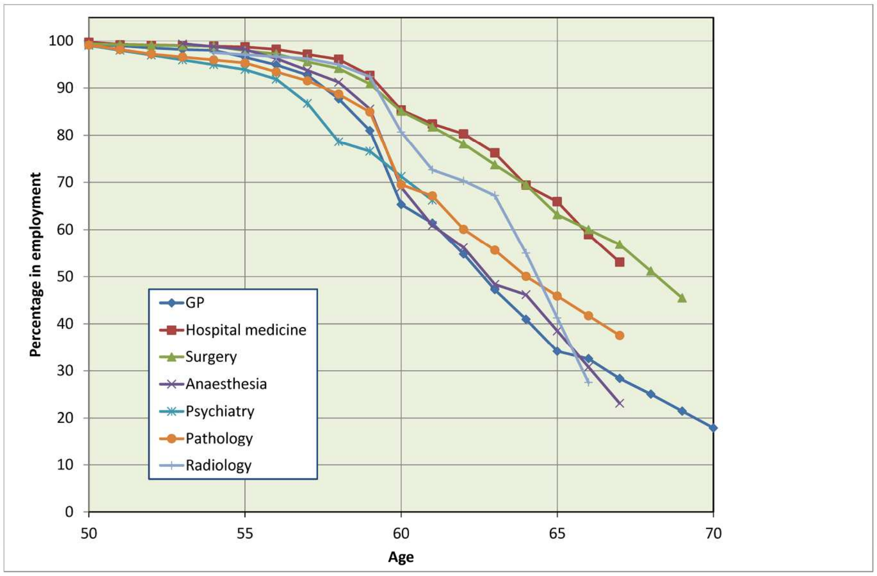

Figure 2: Male 1974 and 1977 graduates still working in medicine, percentages by age: Kaplan-Meier estimates

209x148mm (300 x 300 DPI)

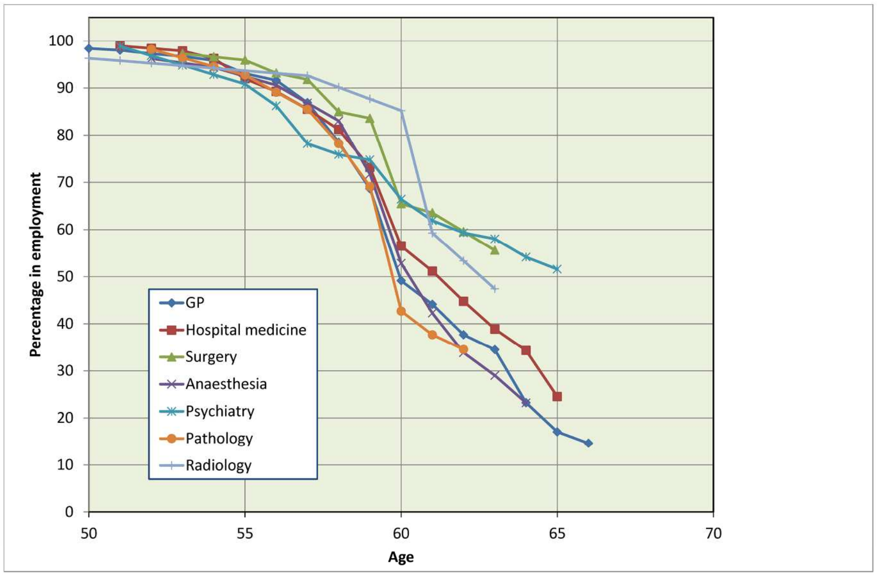

Figure 3: Female 1974 and 1977 graduates still working in medicine, percentages by age: Kaplan-Meier estimates

209x148mm (300 x 300 DPI)

For peer review only

Supplementary Table 1: Employment status at time of survey of the graduates of 1974, by gender and specialty

		Employment status at time of survey									
		Full-time in medicine		Part-time in medicine		'Retired and returned'		Retired		Total	
		N	%	N	%	N	%	N	%	N	%
General practice	Men	40	7.9	62	12.3	97	19.2	306	60.6	505	100.0
	Women	9	4.3	20	9.5	26	12.4	155	73.8	210	100.0
Hospital medicine	Men	46	25.8	19	10.7	59	33.1	54	30.3	178	100.0
	Women	8	9.8	4	4.9	16	19.5	54	65.9	82	100.0
Surgery	Men	40	21.5	11	5.9	76	40.9	59	31.7	186	100.0
	Women	4	15.4	1	3.8	5	19.2	16	61.5	26	100.0
Anaesthesia	Men	9	14.5	5	8.1	18	29.0	30	48.4	62	100.0
	Women	2	11.1	1	5.6	2	11.1	13	72.2	18	100.0
Psychiatry	Men	11	22.9	2	4.2	19	39.6	16	33.3	48	100.0
	Women	1	3.6	2	7.1	13	46.4	12	42.9	28	100.0
Pathology	Men	3	7.3	3	7.3	12	29.3	23	56.1	41	100.0
	Women	1	6.3	1	6.3	5	31.3	9	56.3	16	100.0
Radiology	Men	6	20.7	1	3.4	9	31.0	13	44.8	29	100.0
	Women	1	7.7	0	0.0	4	30.8	8	61.5	13	100.0
Total	Men	155	14.8	103	9.8	290	27.6	501	47.8	1049	100.0
	Women	26	6.6	29	7.4	71	18.1	267	67.9	393	100.0

For peer review only

Supplementary Table 2: Employment status at time of survey of the graduates of 1977, by gender and specialty

		Employment status in 2014									
		Full-time in medicine		Part-time in medicine		'Retired and returned'		Retired		Total	
		N	%	N	%	N	%	N	%	N	%
General practice	Men	97	15.5	87	13.9	184	29.5	256	41.0	624	100.0
	Women	24	7.1	59	17.5	62	18.4	192	57.0	337	100.0
Hospital medicine	Men	91	42.5	18	8.4	66	30.8	39	18.2	214	100.0
	Women	22	20.4	13	12.0	25	23.1	48	44.4	108	100.0
Surgery	Men	111	45.1	19	7.7	70	28.5	46	18.7	246	100.0
	Women	13	28.3	7	15.2	14	30.4	12	26.1	46	100.0
Anaesthesia	Men	23	23.0	6	6.0	31	31.0	40	40.0	100	100.0
	Women	7	20.6	1	2.9	7	20.6	19	55.9	34	100.0
Psychiatry	Men	9	16.7	3	5.6	21	38.9	21	38.9	54	100.0
	Women	8	12.9	7	11.3	25	40.3	22	35.5	62	100.0
Pathology	Men	21	32.8	1	1.6	22	34.4	20	31.3	64	100.0
	Women	3	8.1	4	10.8	6	16.2	24	64.9	37	100.0
Radiology	Men	16	31.4	3	5.9	20	39.2	12	23.5	51	100.0
	Women	5	31.3	4	25.0	3	18.8	4	25.0	16	100.0
Total	Men	368	27.2	137	10.1	414	30.6	434	32.1	1353	100.0
	Women	82	12.8	95	14.8	142	22.2	321	50.2	640	100.0

For peer review only

STROBE 2007 (v4) Statement—Checklist of items that should be included in reports of *cohort studies*

Section/Topic	Item #	Recommendation	Reported on page #
**Title and abstract**	1	(*a*) Indicate the study's design with a commonly used term in the title or the abstract	1
		(*b*) Provide in the abstract an informative and balanced summary of what was done and what was found	2
**Introduction**			
Background/rationale	2	Explain the scientific background and rationale for the investigation being reported	4
Objectives	3	State specific objectives, including any prespecified hypotheses	4
**Methods**			
Study design	4	Present key elements of study design early in the paper	4-5
Setting	5	Describe the setting, locations, and relevant dates, including periods of recruitment, exposure, follow-up, and data collection	4-5
Participants	6	(*a*) Give the eligibility criteria, and the sources and methods of selection of participants. Describe methods of follow-up	4-5
		(*b*) For matched studies, give matching criteria and number of exposed and unexposed	n/a
Variables	7	Clearly define all outcomes, exposures, predictors, potential confounders, and effect modifiers. Give diagnostic criteria, if applicable	4-5
Data sources/ measurement	8*	For each variable of interest, give sources of data and details of methods of assessment (measurement). Describe comparability of assessment methods if there is more than one group	4-5
Bias	9	Describe any efforts to address potential sources of bias	10
Study size	10	Explain how the study size was arrived at	4-5
Quantitative variables	11	Explain how quantitative variables were handled in the analyses. If applicable, describe which groupings were chosen and why	5-9
Statistical methods	12	(*a*) Describe all statistical methods, including those used to control for confounding	5-9
		(*b*) Describe any methods used to examine subgroups and interactions	5-9
		(*c*) Explain how missing data were addressed	n/a
		(*d*) If applicable, explain how loss to follow-up was addressed	4
		(*e*) Describe any sensitivity analyses	n/a
**Results**			

For peer review only

Participants	13*	(a) Report numbers of individuals at each stage of study—eg numbers potentially eligible, examined for eligibility, confirmed eligible, included in the study, completing follow-up, and analysed	6
		(b) Give reasons for non-participation at each stage	6
		(c) Consider use of a flow diagram	n/a
Descriptive data	14*	(a) Give characteristics of study participants (eg demographic, clinical, social) and information on exposures and potential confounders	6-9
		(b) Indicate number of participants with missing data for each variable of interest	6-9
		(c) Summarise follow-up time (eg, average and total amount)	4
Outcome data	15*	Report numbers of outcome events or summary measures over time	n/a
Main results	16	(*a*) Give unadjusted estimates and, if applicable, confounder-adjusted estimates and their precision (eg, 95% confidence interval). Make clear which confounders were adjusted for and why they were included	n/a
		(*b*) Report category boundaries when continuous variables were categorized	n/a
		(*c*) If relevant, consider translating estimates of relative risk into absolute risk for a meaningful time period	n/a
Other analyses	17	Report other analyses done—eg analyses of subgroups and interactions, and sensitivity analyses	n/a
**Discussion**			
Key results	18	Summarise key results with reference to study objectives	6-9
**Limitations**			
Interpretation	20	Give a cautious overall interpretation of results considering objectives, limitations, multiplicity of analyses, results from similar studies, and other relevant evidence	6-11
Generalisability	21	Discuss the generalisability (external validity) of the study results	10-11
**Other information**			
Funding	22	Give the source of funding and the role of the funders for the present study and, if applicable, for the original study on which the present article is based	12

*Give information separately for cases and controls in case-control studies and, if applicable, for exposed and unexposed groups in cohort and cross-sectional studies.

Note: An Explanation and Elaboration article discusses each checklist item and gives methodological background and published examples of transparent reporting. The STROBE checklist is best used in conjunction with this article (freely available on the Web sites of PLoS Medicine at http://www.plosmedicine.org/, Annals of Internal Medicine at http://www.annals.org/, and Epidemiology at http://www.epidem.com/). Information on the STROBE Initiative is available at www.strobe-statement.org.

For peer review only

BMJ Open

Retirement ages of senior UK doctors: national surveys of the medical graduates of 1974 and 1977

Journal:	*BMJ Open*
Manuscript ID	bmjopen-2018-022475.R1
Article Type:	Research
Date Submitted by the Author:	22-May-2018
Complete List of Authors:	Smith, Fay; University of Oxford, Nuffield Department of Population Health<br>Goldacre, Michael; University of Oxford, Nuffield Department of Population Health<br>Lambert, Trevor; University of Oxford, Nuffield Department of Population Health
**Primary Subject Heading**:	Health services research
Secondary Subject Heading:	Health services research, Medical education and training
Keywords:	MEDICAL EDUCATION & TRAINING, Physicians, workforce, retirement

SCHOLARONE™
Manuscripts

Retirement ages of senior UK doctors: national surveys of the medical graduates of 1974 and 1977

Short title: Retirement ages of senior UK doctors

Fay Smith PhD[1], Michael J Goldacre FFPH[1] , Trevor W Lambert MSc[1]

[1]UK Medical Careers Research Group, Unit of Health-Care Epidemiology, Nuffield Department of Population Health, University of Oxford, Old Road Campus, Oxford OX3 7LF

Corresponding author: Trevor Lambert (trevor.lambert@dph.ox.ac.uk; Telephone: 01865 289389)

Word count: 2346 words.

For peer review only

ABSTRACT

Objective: To report on retirement ages of two cohorts of senior doctors in the latter stages of their careers.

Design: Questionnaires sent in 2014 to all medical graduates of 1974 and 1977.

Setting: UK.

Participants: 3695 UK medical graduates.

Main outcome measures: Retirement status by age at the time of the survey, and age at retirement if retired. Planned retirement ages and retirement plans if not retired.

Results: Of contactable doctors, 85% responded. 43.7% of all responding doctors had fully retired, 25.9% had 'retired and returned' for some medical work, 18.3% had not retired and were working full-time in medicine, 10.7% had not retired and were working part-time in medicine, and 1.4% were either doing non-medical work or did not give details of their employment status. The average actual retirement age (including those who had retired but subsequently returned) was 59.6 years (men 59.9, women 58.9). Psychiatrists (58.3) and GPs (59.5) retired at a slightly younger age than radiologists (60.4), surgeons (60.1) and hospital specialists (60.0). More GPs (54%) than surgeons (26%) or hospital medical specialists (34%) were fully retired, and there were substantial variations in retirement rates in other specialties. Sixty-three percent of women GPs were fully retired.

Conclusions: Gender and specialty differences in retirement ages were apparent and are worthy of qualitative study to establish underlying reasons in those specialties where earlier retirement is more common. There is a general societal expectation that people will retire at increasingly elderly ages; but the doctors in this national study retired relatively young.

[252 words]

For peer review only - http://bmjopen.bmj.com/site/about/guidelines.xhtml

Strengths and limitations of this study

- This is a large scale study of graduates from all UK medical schools.
- The study has a very high response rate (85%) for a questionnaire study with voluntary participation.
- The study succeeded in differentiating between doctors who had fully retired and doctors who had taken formal retirement and returned in a medical capacity.
- However there is a possibility of non-responder bias, in that retired doctors may have been less likely than doctors who had not retired to be easy to contact or inclined to respond.

For peer review only

For peer review only - http://bmjopen.bmj.com/site/about/guidelines.xhtml

INTRODUCTION

In the United Kingdom (UK), as in many other countries, as average life expectancy increases, retirement age and pensionable age will have to increase too. The World Economic Forum has reported that a baby born in 2017 can expect to live to over 100, and more immediately, retirement systems that were designed to support a retirement of 10-15 years will have to change to prepare for a "seismic shift" as without substantially increasing retirement ages, individuals would eventually be spending more time retired than working.[1] Already changes are happening in the UK: for example the state pension age is rising to 66 in 2020 and to 67 by 2028.[2]

Against this background, and the trend to work longer to ensure financial security in old age, there are data which suggest that older doctors, both in the UK and in other countries, far from working longer, are in fact retiring early or reducing their working hours in increasing numbers in recent years.[3 4] A recent survey of consultant physicians in the UK found that their average intended age of retirement was 62 years, and that 72% of these doctors did not intend to work beyond retirement age.[5] Early retirement of doctors has the major consequence for health-care of early loss of doctors from the medical workforce.

In 2014, 20% of UK consultants and 21% of General Practitioners (GPs) were aged 55 or over.[6] The median retirement age for GPs in the UK was 58 for women and 60 for men, between the years 2008 and 2011.[7]

The aim of this paper is to report on the retirement status and ages, and retirement intentions, of two cohorts of senior doctors who graduated in 1974 and 1977 whom we surveyed in 2014 when they had median ages of 64 and 61 years old respectively. We compared the replies of men and women, of those in the two cohorts, and of those working in different career specialties.

METHODS

The UK Medical Careers Research Group surveyed the UK medical graduates of 1974 and 1977. This was our last planned survey in career-long studies of the professional choices and work trajectories of these cohorts.[8-14] We sent postal and web-based questionnaires to these senior doctors in 2014 using current contact details provided to us by the General Medical Council. Up to four reminders were sent to non-respondents. Further details of the methodology are available elsewhere.[15]

For peer review only - http://bmjopen.bmj.com/site/about/guidelines.xhtml

The surveys sent to both cohorts were identical and comprised structured, 'closed' questions and statements. Doctors were asked to indicate their current employment status, choosing from seven categories (working in medicine full-time or part-time, working outside medicine full-time or part-time, retired, 'retired and returned' for some medical work, or other). Doctors were then asked to respond to questions about their retirement age (if they had retired), or their retirement plans (if still working; see Box 1).

Box 1: Questions and statements presented to respondents

Asked of	Focus	Question/Statement
Retirees and doctors who had 'retired and returned'	Retirement age	How old were you when you retired?
Doctors still working in medicine (full or part-time)	Retirement plans	At what age do you plan to retire?<br><br>Do you plan to reduce your time commitments to your work before retiring? [Yes, I have already done so; Yes, in the future; No; Don't know]

We allocated a career specialty to each respondent using their recorded job history as reported to us in successive surveys and additional information about their specialist registration with the GMC as reported to us by the doctors in these surveys. For a small number of respondents we were unable to allocate a single career specialty, either because we did not have sufficient data about the doctor's career, or because the doctor had worked in different specialties during their career. The career specialty allocation allowed us to analyse the responses and employment status of doctors in different specialties. Respondents were then grouped for analysis into seven groups: general practice, hospital medicine (comprising the hospital physician specialties and paediatrics), surgery (comprising the surgical specialties, emergency medicine, obstetrics and gynaecology, and clinical oncology), anaesthesia, psychiatry, pathology, and radiology.

The replies were analysed using χ^2 tests and Mann-Whitney U tests to explore differences between male and female doctors, between cohorts and between doctors working in different specialties.

During ethical approval we obtained permission to approach doctors for participation and to take their response as an indicator of informed consent to participate in the studies.

For peer review only - http://bmjopen.bmj.com/site/about/guidelines.xhtml

Patient and Public Involvement: Patients were not involved in the design or any aspect of the study, by agreement with the funding body, since the study did not involve any medical or patient data. Results of the study are published in various papers in the peer-reviewed literature, and summary reports of our survey work are on our website at www.ndph.ox.ac.uk

For peer review only

For peer review only - http://bmjopen.bmj.com/site/about/guidelines.xhtml

RESULTS

Response rates

We were able to contact 4369 of the original 5482 UK medical graduates from 1974 and 1977 (Table 1). The aggregated response rate from both cohorts was 84.6% (3695/4369). To maximise response, at the end of the survey period non-responders were given the option of completing a shortened questionnaire: 98 did so, and because of the curtailed information obtained from them, they could not be included in some of the analysis below.

Table 1: UK doctors who graduated in 1974 and 1977: response to survey, and career status of respondents

TARGET GROUPING	Year of graduation 1974	Year of graduation 1977	Total
GRADUATION COHORT	2347 (100%)	3135 (100%)	5482 (100%)
Known to be deceased	*100 (4.3%)*	*110 (3.5%)*	*210 (3.8%)*
Declined to participate	*20 (0.9%)*	*50 (1.6%)*	*70 (1.3%)*
Uncontactable	*415 (17.7%)*	*418 (13.3%)*	*833 (15.2%)*
Contacted	*1812 (77.2%)*	*2557 (81.6%)*	*4369 (79.7%)*
CONTACTABLE DOCTORS	1812 (100%)	2557 (100%)	4369 (100%)
Did not respond	*267 (14.7%)*	*407 (15.9%)*	*674 (15.4%)*
Responded in brief	*47 (2.6%)*	*51 (2.0%)*	*98 (2.2%)*
Responded in full	*1498 (82.7%)*	*2099 (82.1%)*	*3597 (82.3%)*
RESPONDENTS	1545 (100%)	2150 (100%)	3695 (100%)
Working full-time in medicine	*195 (12.6%)*	*483 (22.5%)*	*678 (18.3%)*
Working part-time in medicine	*145 (9.4%)*	*252 (11.7%)*	*397 (10.7%)*
Working full-time outside medicine	*6 (0.4%)*	*10 (0.5%)*	*16 (0.4%)*
Working part-time outside medicine	*7 (0.5%)*	*9 (0.4%)*	*16 (0.4%)*
Retired, not now working in medicine	*804 (52.0%)*	*810 (37.7%)*	*1614 (43.7%)*
Retired, and 'returned' for some medical work	*380 (24.6%)*	*577 (26.8%)*	*957 (25.9%)*
Other / no reply	*8 (0.5%)*	*9 (0.4%)*	*17 (0.5%)*

Employment status in 2014

In 2014, 43.7% of all responding doctors had retired, 25.9% had 'retired and returned' for some medical work, 18.3% were working full-time in medicine, 10.7% were working part-time in medicine, and 1.3% were either doing non-medical work or did not give details of their employment status (Table 1). Having graduated three years earlier than the 1977 graduates, more doctors from the 1974 cohort (52.0%) had retired than from the 1977 cohort (37.7%).

In further analysis, we excluded doctors whose career specialty (see Method) was unknown, or who were working outside medicine, or who did not declare their employment status, and analysed the employment status in 2014 of the 3435 respondents remaining, by gender and specialty grouping (Table 2).

For peer review only - http://bmjopen.bmj.com/site/about/guidelines.xhtml

Table 2: Career specialty, and employment status at time of survey, of graduates of 1974 and 1977

	Employment status				
	Full-time in medicine	Part-time in medicine	'Retired and returned'	Retired	Total N (100%)
	%	%	%	%	%
Total	18.4	10.6	26.7	44.3	3435
General practice	10.1	13.6	22.0	54.2	1676
Hospital medicine	28.7	9.3	28.5	33.5	582
Surgery	33.3	7.5	32.7	26.4	504
Anaesthesia	19.2	6.1	27.1	47.7	214
Psychiatry	15.1	7.3	40.6	37.0	192
Pathology	17.7	5.7	28.5	48.1	158
Radiology	25.7	7.3	33.0	33.9	109
Women	10.5	12.0	20.6	56.9	1033
General practice	6.0	14.4	16.1	63.4	547
Hospital medicine	15.8	8.9	21.6	53.7	190
Surgery	23.6	11.1	26.4	38.9	72
Anaesthesia	17.3	3.8	17.3	61.5	52
Psychiatry	10.0	10.0	42.2	37.8	90
Pathology	7.5	9.4	20.8	62.3	53
Radiology	20.7	13.8	24.1	41.4	29
Men	21.8	10.0	29.3	38.9	2402
General practice	12.1	13.2	24.9	49.8	1129
Hospital medicine	34.9	9.4	31.9	23.7	392
Surgery	35.0	6.9	33.8	24.3	432
Anaesthesia	19.8	6.8	30.2	43.2	162
Psychiatry	19.6	4.9	39.2	36.3	102
Pathology	22.9	3.8	32.4	41.0	105
Radiology	27.5	5.0	36.3	31.3	80

Percentages are row percentages.
Excludes 32 respondents working in non-medical jobs and 228 whose employment status and/or career specialty was unknown.
See Method for explanation of career specialty grouping.
For detailed specialty breakdown in each cohort, with numbers and percentages, see Supplementary Tables 1 and 2.

Taking men and women together, employment status and career specialty were strongly related (χ^2_{18}=324.1, p<.001): the most notable specialty differences were that more general practitioners (54%), and fewer surgeons (26%) and hospital medical specialists (34%), were fully retired compared to the overall average; and fewer general practitioners (10%) and more surgeons (33%) and hospital medical specialists (29%) were working full-time compared to the overall average (based on analysis of adjusted residuals).

For peer review only - http://bmjopen.bmj.com/site/about/guidelines.xhtml

Comparing specialties separately for men and women (Table 2), the specialty differences in employment status for men were significant (χ^2_{18}=2241.7, p<.001), and in the same specialties as the total. For women, the specialty differences in employment status were also significant (χ^2_{18}=84.3, p<.001); but the percentage of women hospital medical specialists who were retired (54%) was much closer to the percentage of women general practitioners (63%) who were retired, than was the case for the men (24% vs 50%).

Supplementary tables 1 and 2 show results for the 1974 and 1977 cohorts separately. In many cases the numbers in individual specialties are too small for meaningful statistical comparisons to be made.

Age at retirement

Figure 1 shows the percentages of men and women respondents in each cohort who had not retired and who were working in medicine at each year of age. (The figure treats 'retired and returned' as not working in medicine for this purpose). Log rank tests showed that the male-female difference was significant in the 1974 cohort (χ^2_1=93.9, p<.001) and in the 1977 cohort (χ^2_1=78.8, p<.001): at any age the percentage of women who were retired was larger than that of men. Within gender, the two cohorts followed similar patterns of retirement by age (women χ^2_1=0.3, p=.57; men χ^2_1=0.7, p=.39).

Figures 2 and 3 show the data for men and women separately, subdivided by career specialty, based on the seven groups previously defined (see Method). The cohorts have been combined but the gender separation has been retained, in view of the results from Figure 1. Specialty differences were more marked for men (χ^2_1=130.7, Figure 2) than for women (χ^2_1=25.1, Figure 3), both p<.001. Within each specialty, the gender difference was significant with p<.001 for general practice and hospital medicine, and was borderline significant for surgery, anaesthesia, and pathology (in each case with higher percentages of women than men having retired): there was no gender difference for psychiatry or radiology (both p>.05).

The average actual retirement age (including those who had retired but subsequently returned) was 59.6 years (59.9 men, 58.9 women; 60.4 1974 cohort, 58.9 1977 cohort). Psychiatrists (58.3) and GPs (59.5) retired at a slightly younger age than radiologists (60.4), surgeons (60.1) and hospital specialists (60.0) (χ^2_6=67.8, p<.001; Kruskal Wallis test).

For peer review only - http://bmjopen.bmj.com/site/about/guidelines.xhtml

Doctors who had not retired and who were still working in medicine: planned retirement ages

The average planned retirement age of doctors still working was 65.7 years (66.0 men, 64.7 women; 67.4 1974 cohort, 64.9 1977 cohort; 65.9 full-time, 65.3 part-time). Psychiatrists still working planned to retire at an older age (67.4) than doctors in other specialties: anaesthetists (64.5), pathologists (64.9), GPs (65.2) and radiologists (65.2) (χ^2_6=26.0, p<.001; Kruskal Wallis test).

Doctors who had not retired and who were still working: retirement plans

When asked 'Do you plan to reduce your time commitments to your work before retiring?' (38.2%) of doctors who were still working answered 'Yes, I have already done so', 29.2% answered 'Yes, in the future', 22.2% answered 'No', and 10.4% answered 'Don't know'. More men (32.2%) than women (19.5%) answered 'Yes, in the future' (χ^2_4=21.5, p<.001). There were significant differences between specialties (χ^2_{18}=69.9, p<.001). More GPs (53.1%) answered 'Yes, I have already done so' than surgeons (29.7%) and doctors in hospital medicine (29.3%).

DISCUSSION

Main findings

Given that these doctors were predominantly only in their early sixties, the level of retirement was surprisingly high given societal changes in life expectancy and retirement planning. 'Retiring and returning', often to a less demanding or less time-committed post, and substantial levels of part-time working among the remainder, meant that there remained only a small minority of these highly experienced doctors working full-time in medicine.

Specialty differences in retirement levels at the time of the survey were seen for both men and women. They were more pronounced for men, with male hospital doctors having low levels of retirement compared to other men, or women. This may reflect gender roles and expectation in this older generation, factors which may not apply to the same extent to younger generations when they reach the equivalent stages in their careers.

Retirement profiles by age showed similar results and, additionally, in general practice, the hospital medical specialties, surgery, anaesthesia, and pathology, the proportion of women who had retired increased more with increasing age than was the case for men.

For peer review only - http://bmjopen.bmj.com/site/about/guidelines.xhtml

Among those who had retired early, changes in the work environment and in personal circumstances were often contributory factors.

GPs who were still working were more inclined to have planned work reductions than their hospital colleagues.

Strengths and limitations

This is large scale study of graduates from all UK medical schools. The study has a very high response rate (85%) for a questionnaire study with voluntary participation. However there is a possibility of non-responder bias.

In 2017, in the UK 47% of the registered UK medical workforce were women: of doctors aged under 30 years (13% of the whole workforce), 62% were women.[16] By contrast, in our study women constituted only 30% of the 1974/1977 cohorts. We cannot judge whether younger cohorts, with higher percentages of women, will eventually show similar profiles of retirement to the subjects in our study.

Comparison with existing literature

The doctors we surveyed retired, on average, aged 60 – two years earlier than the average intended retirement age given by consultants in a UK census conducted in 2014-5.[5] The UK census however comprises consultants who are older than the 1974 and 1977 cohorts we studied. On 1 February 2018 Pulse Magazine reported that analysis of figures received for NHS Business Services Authority under a Freedom of Information request showed that 62% of GPs claiming their pension for the first time in 2016/17 were under 60 years old, compared with 33% in 2011/12. A review of studies which reported both intended and actual retirement ages found the two ages to be similar.[17] As mentioned in the Introduction, there is a general societal trend towards later retirement (which contrasts with our finding of doctors' earlier retirement), with UK women in particular working longer as the state pension age increases.[18-20] An earlier analysis of these cohorts, reporting a median age of 64 for those who retired in 2014, found that 27% had retired earlier than they had originally intended; and, of those who had retired, their main reasons for retirement were 'increased time for leisure/other interests' and 'pressure of work'.[21] A systematic review of physician retirement identified burnout and excessive workload as the main reasons for early retirement.[17]

For peer review only - http://bmjopen.bmj.com/site/about/guidelines.xhtml

We found that more GPs (54%) than doctors in other specialties were retired, and 63% of women GPs were retired. Factors influencing GPs' intentions to leave general practice include workload intensity, bureaucracy, appraisal and revalidation.[22-24] Research in Australia of doctors aged over 55 found that psychiatrists and GPs were less likely than doctors in other specialties to say that they intended to retire,[25] and research in Canada found no difference in retirement age between specialties.[26] In the UK, GPs are reported to have had higher levels of burnout than doctors in other specialties.[27]

Implications / conclusions

Our findings demonstrate the scale of loss of doctors from the workforce as a result of retirement in the early 60s. Premature loss of experienced doctors in late career has an adverse impact on workforce capacity and it needs to be better understood. Nonetheless, it is difficult to untangle the motivations of doctors as they consider and plan the reality and timing of their retirement. Every retirement, like every medical career, will be individual and will be the outcome of a number of considerations related to work, home life and leisure. In these cohorts, women are retiring younger than men and there was substantial variation by specialty. There is scope for further study of underlying reasons for early retirement, and in particular to address the question of whether those attracted to some specialties are more likely than others to have characteristics which predispose them to early retirement, or whether there are inherent issues in some specialties which render early retirement more attractive. If the latter, policy amendments to ameliorate these issues may reduce workforce losses consequent to early retirement.

[2346 words]

For peer review only

For peer review only - http://bmjopen.bmj.com/site/about/guidelines.xhtml

Conflict of interests: All authors have completed the Unified Competing Interest form at www.icmje.org/coi_disclosure.pdf (available on request from the corresponding author) and all authors want to declare: (1) financial support for the submitted work from the policy research programme, Department of Health. All authors also declare: (2) no financial relationships with commercial entities that might have an interest in the submitted work; (3) no spouses, partners, or children with relationships with commercial entities that might have an interest in the submitted work; (4) no non-financial interests that may be relevant to the submitted work.

Funding: The report is based on independent research commissioned and funded by the NIHR Policy Research Programme (project number 016/0118). The views expressed in the publication are those of the author(s) and not necessarily those of the NHS, the NIHR, the Department of Health, its arm's length bodies or other government departments.

Data sharing statement: It may be possible for the authors to make tabulated data, produced in the course of this work but not included in the paper, available to interested readers on request.

Ethical approval: National Research Ethics Service, following referral to the Brighton and Mid-Sussex Research Ethics Committee in its role as a multi-centre research ethics committee (ref 04/Q1907/48 amendment Am02 March 2015).

Guarantor: All authors are guarantors.

Contributorship: TL and MJG designed and conducted the surveys. FS performed the analysis and wrote the first drafts of the paper. All authors contributed to further drafts and all approved the final version.

Acknowledgements: We thank Shelly Lachish for early work on the paper and Janet Justice and Alison Stockford for data entry. We are very grateful to all the doctors who participated in the surveys.

MeSH terms: Physicians; workforce; medical; retirement.

What this paper adds

For peer review only - http://bmjopen.bmj.com/site/about/guidelines.xhtml

What is already known

- There is a societal trend for retirement ages and pensionable ages to increase with increased life expectancy
- However, some studies have suggested that doctors are retiring early in increasing numbers, or are reducing their work commitments in the later stages of their careers

What this study adds

- Many doctors in our study had retired completely by their early 60s, or had ‘retired and returned’ to a less demanding or time-committed post
- However, there was substantial variation by specialty and by gender

For peer review only - http://bmjopen.bmj.com/site/about/guidelines.xhtml

REFERENCES

1. World Economic Forum. We'll Live to 100 – How Can We Afford It? Geneva: World Economic Forum, 2017.

2. Department for Work and Pensions. State Pension age timetable. London: Department for Work and Pensions, 2013.

3. Royal College of Physicians. Census of consultant physicians and higher specialty trainees in the UK 2016-17. London: Royal Colleges of Physicians, 2017.

4. Joyce CM, Wang WC, McDonald HM. Retirement patterns of Australian doctors aged 65 years and older. Aust. Health Rev. 2015;**39**(5):582-87 doi: 10.1071/AH14176 [published Online First: 22 June 2015].

5. Royal College of Physicians. Census of consultant physicians and higher specialty trainees in the UK 2014-15. London: Royal Colleges of Physicians, 2016.

6. British Medical Association. 2014 UK Medical Workforce Briefing. London: British Medical Association, 2014.

7. Centre for Workforce Intelligence. In-depth review of the general practitioner workforce. London: Centre for Workforce Intelligence, 2014.

8. Parkhouse J, McLaughlin C. Career preferences of doctors graduating in 1974. Br. Med. J. 1976;**2**(6036):630-2 1976/09/11].

9. Parkhouse J, Palmer MK, Hambleton BA. Career preferences of doctors qualifying in the United Kingdom in 1977. Health Trends 1979;**11**(2):35-7.

10. Parkhouse J, Campbell MG, Parkhouse HF. Careers of doctors qualifying in Britain in 1974: a report on their employment status in 1979. Br. Med. J. (Clin. Res. Ed). 1982;**285**(6340):522-6 1982/08/14].

11. Davidson JM, Lambert TW, Goldacre MJ. Career pathways and destinations 18 years on among doctors who qualified in the United Kingdom in 1977: Postal questionnaire survey. Br. Med. J. 1998;**317**(7170):1425-28.

12. Taylor K, Lambert T, Goldacre M. Future career plans of a cohort of senior doctors working in the National Health Service. J. R. Soc. Med. 2008;**101**(4):182-90.

13. Taylor K, Lambert T, Goldacre M. Career destinations, job satisfaction and views of the UK medical qualifiers of 1977. J. R. Soc. Med. 2008;**101**(4):191-200.

14. Goldacre MJ, Laxton L, Lambert TW. Medical graduates' early career choices of specialty and their eventual specialty destinations: UK prospective cohort studies. BMJ 2010;**341**(7764):139 doi: 10.1136/bmj.c3199 [published Online First: 06 July 2010].

For peer review only - http://bmjopen.bmj.com/site/about/guidelines.xhtml

15. Goldacre M, Lambert T. Participation in medicine by graduates of medical schools in the United Kingdom up to 25 years post graduation: National cohort surveys. Acad. Med. 2013;**88**(5):699-709 doi: 10.1097/ACM.0b013e31828b364f [published Online First: 01 May 2013].
16. General Medical Council. The state of medical education and practice in the UK report: 2017. London: GMC, 2017.
17. Silver MP, Hamilton AD, Biswas A, et al. A systematic review of physician retirement planning. Human Resources for Health 2016;**14**(1):67 doi: 10.1186/s12960-016-0166-z [published Online First: 15 November 2016].
18. Emmerson C, Heald K, Hood A. The Changing Face of Retirement: Future Patterns of Work, Health, Care and Income among the Older Population. London: Institute for Fiscal Studies, 2014.
19. Houses of Parliament: parliamentary office of science and technology. An Ageing Workforce. London: Houses of Parliament, 2011.
20. Demou E, Bhaskar A, Xu T, et al. Health, lifestyle and employment beyond state-pension age. BMC Public Health 2017;**17**(1) doi: 10.1186/s12889-017-4957-5 [published Online First: 20 December 2017].
21. Smith F, Lachish S, Goldacre MJ, et al. Factors influencing the decisions of senior UK doctors to retire or remain in medicine: national surveys of the UK-trained medical graduates of 1974 and 1977. BMJ open 2017;**7**(9) doi: 10.1136/bmjopen-2017-017650 [published Online First: 1 November 2017].
22. Dale J, Potter R, Owen K, et al. The general practitioner workforce crisis in England: A qualitative study of how appraisal and revalidation are contributing to intentions to leave practice. BMC Family Practice 2016;**17**(1) doi: 10.1186/s12875-016-0489-9 [published Online First: 20 July 2016].
23. Dale J, Potter R, Owen K, et al. Retaining the general practitioner workforce in England: What matters to GPs? A cross-sectional study. BMC Family Practice 2015;**16**(1) doi: 10.1186/s12875-015-0363-1 [published Online First: 16 October 2015].
24. Sansom A, Calitri R, Carter M, et al. Understanding quit decisions in primary care: A qualitative study of older GPs. BMJ Open 2016;**6**(2):e010592 doi: 10.1136/bmjopen-2015-010592 [published Online First: 19 February 2016].
25. Wijeratne C, Earl JK, Peisah C, et al. Professional and psychosocial factors affecting the intention to retire of Australian medical practitioners. Med. J. Aust. 2017;**206**(5):209-14 doi: 10.5694/mja16.00883 [published Online First: 20 March 2017].

For peer review only - http://bmjopen.bmj.com/site/about/guidelines.xhtml

26. Hedden L, Lavergne MR, McGrail KM, et al. Patterns of physician retirement and pre-retirement activity: A population-based cohort study. CMAJ 2017;**189**(49):E1517-E23 doi: 10.1503/cmaj.170231 [published Online First: 11 December 2017].
27. Halliday L, Walker A, Vig S, et al. Grit and burnout in UK doctors: A cross-sectional study across specialties and stages of training. Postgrad. Med. J. 2017;**93**(1101):389-94 doi: 10.1136/postgradmedj-2015-133919 [published Online First: 18 November 2016].

For peer review only

For peer review only - http://bmjopen.bmj.com/site/about/guidelines.xhtml

FIGURE CAPTIONS

Figure 1: Percentage of 1974 and 1977 graduates who had not retired and who were working in medicine, by age: Kaplan-Meier estimates

Short legend: Doctors working in medicine, by age

Figure 2: Male 1974 and 1977 graduates still working in medicine, percentages by age: Kaplan-Meier estimates

Short legend: Male doctors working in medicine, by specialty

Figure 3: Female 1974 and 1977 graduates still working in medicine, percentages by age: Kaplan-Meier estimates

Short legend: Female doctors working in medicine, by specialty

For peer review only

For peer review only - http://bmjopen.bmj.com/site/about/guidelines.xhtml

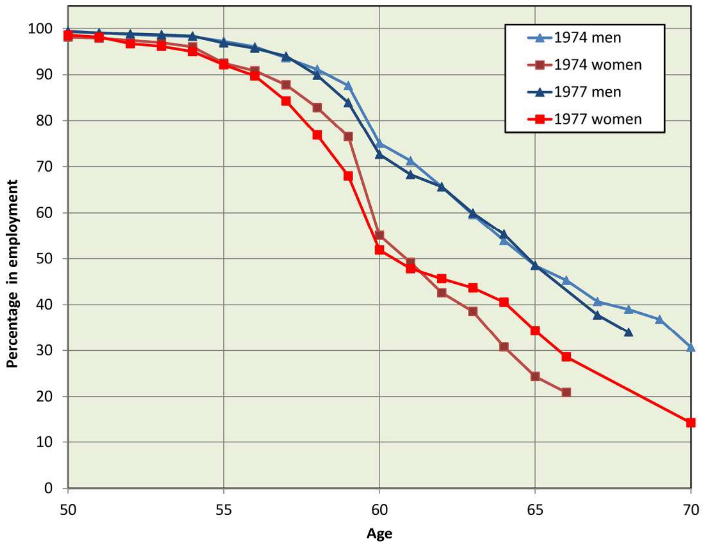

Figure 1: Percentage of 1974 and 1977 graduates who had not retired and who were working in medicine, by age: Kaplan-Meier estimates

Short legend: Doctors working in medicine, by age

209x148mm (300 x 300 DPI)

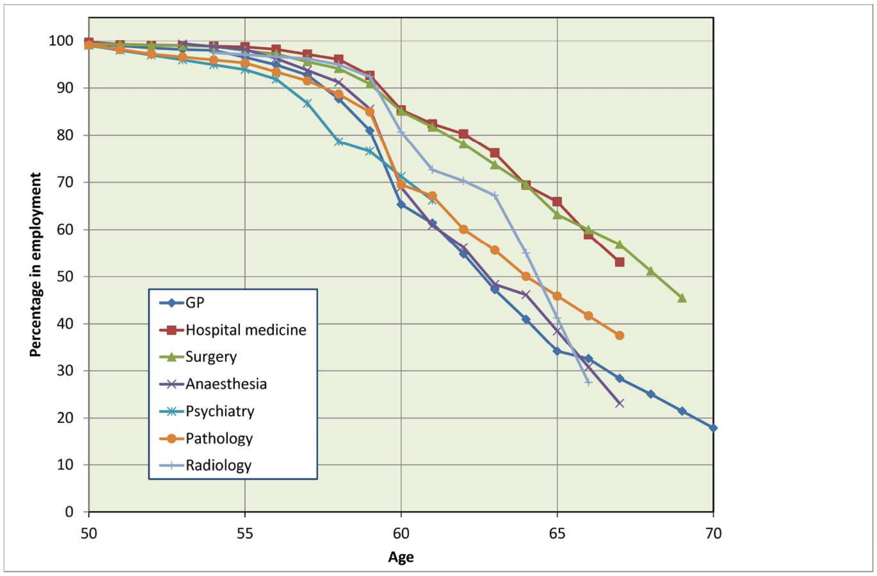

Figure 2: Male 1974 and 1977 graduates still working in medicine, percentages by age: Kaplan-Meier estimates

Short legend: Male doctors working in medicine, by specialty

209x148mm (300 x 300 DPI)

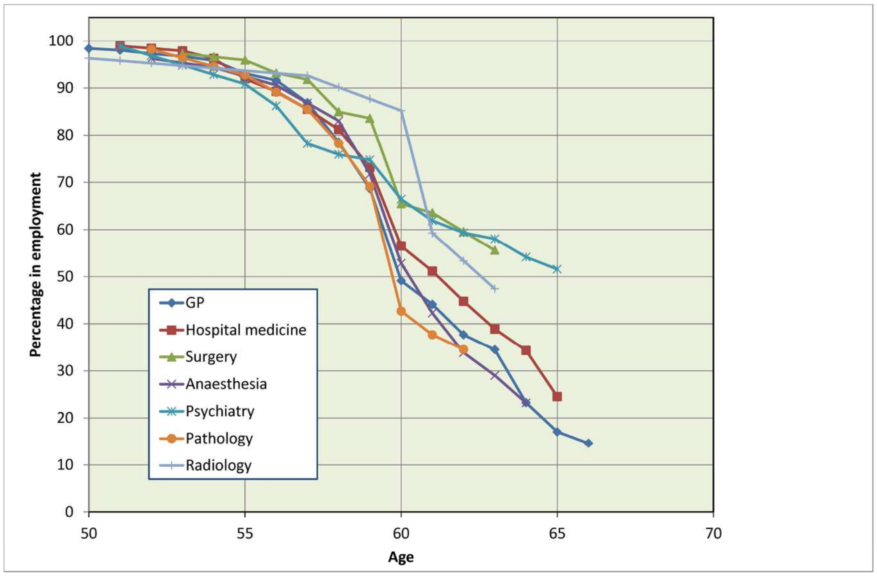

Figure 3: Female 1974 and 1977 graduates still working in medicine, percentages by age: Kaplan-Meier estimates

Short legend: Female doctors working in medicine, by specialty

209x148mm (300 x 300 DPI)

only

Supplementary Table 1: Employment status at time of survey of the graduates of 1974, by gender and specialty

		Employment status at time of survey									
		Full-time in medicine		Part-time in medicine		'Retired and returned'		Retired		Total	
		N	%	N	%	N	%	N	%	N	%
General practice	Men	40	7.9	62	12.3	97	19.2	306	60.6	505	100.0
	Women	9	4.3	20	9.5	26	12.4	155	73.8	210	100.0
Hospital medicine	Men	46	25.8	19	10.7	59	33.1	54	30.3	178	100.0
	Women	8	9.8	4	4.9	16	19.5	54	65.9	82	100.0
Surgery	Men	40	21.5	11	5.9	76	40.9	59	31.7	186	100.0
	Women	4	15.4	1	3.8	5	19.2	16	61.5	26	100.0
Anaesthesia	Men	9	14.5	5	8.1	18	29.0	30	48.4	62	100.0
	Women	2	11.1	1	5.6	2	11.1	13	72.2	18	100.0
Psychiatry	Men	11	22.9	2	4.2	19	39.6	16	33.3	48	100.0
	Women	1	3.6	2	7.1	13	46.4	12	42.9	28	100.0
Pathology	Men	3	7.3	3	7.3	12	29.3	23	56.1	41	100.0
	Women	1	6.3	1	6.3	5	31.3	9	56.3	16	100.0
Radiology	Men	6	20.7	1	3.4	9	31.0	13	44.8	29	100.0
	Women	1	7.7	0	0.0	4	30.8	8	61.5	13	100.0
Total	Men	155	14.8	103	9.8	290	27.6	501	47.8	1049	100.0
	Women	26	6.6	29	7.4	71	18.1	267	67.9	393	100.0

For peer review only

Supplementary Table 2: Employment status at time of survey of the graduates of 1977, by gender and specialty

		Employment status in 2014									
		Full-time in medicine		Part-time in medicine		'Retired and returned'		Retired		Total	
		N	%	N	%	N	%	N	%	N	%
General practice	Men	97	15.5	87	13.9	184	29.5	256	41.0	624	100.0
	Women	24	7.1	59	17.5	62	18.4	192	57.0	337	100.0
Hospital medicine	Men	91	42.5	18	8.4	66	30.8	39	18.2	214	100.0
	Women	22	20.4	13	12.0	25	23.1	48	44.4	108	100.0
Surgery	Men	111	45.1	19	7.7	70	28.5	46	18.7	246	100.0
	Women	13	28.3	7	15.2	14	30.4	12	26.1	46	100.0
Anaesthesia	Men	23	23.0	6	6.0	31	31.0	40	40.0	100	100.0
	Women	7	20.6	1	2.9	7	20.6	19	55.9	34	100.0
Psychiatry	Men	9	16.7	3	5.6	21	38.9	21	38.9	54	100.0
	Women	8	12.9	7	11.3	25	40.3	22	35.5	62	100.0
Pathology	Men	21	32.8	1	1.6	22	34.4	20	31.3	64	100.0
	Women	3	8.1	4	10.8	6	16.2	24	64.9	37	100.0
Radiology	Men	16	31.4	3	5.9	20	39.2	12	23.5	51	100.0
	Women	5	31.3	4	25.0	3	18.8	4	25.0	16	100.0
Total	Men	368	27.2	137	10.1	414	30.6	434	32.1	1353	100.0
	Women	82	12.8	95	14.8	142	22.2	321	50.2	640	100.0

For peer review only

STROBE 2007 (v4) Statement—Checklist of items that should be included in reports of *cohort studies*

Section/Topic	Item #	Recommendation	Reported on page #
**Title and abstract**	1	(*a*) Indicate the study's design with a commonly used term in the title or the abstract	1
		(*b*) Provide in the abstract an informative and balanced summary of what was done and what was found	2
**Introduction**			
Background/rationale	2	Explain the scientific background and rationale for the investigation being reported	4
Objectives	3	State specific objectives, including any prespecified hypotheses	4
**Methods**			
Study design	4	Present key elements of study design early in the paper	4-5
Setting	5	Describe the setting, locations, and relevant dates, including periods of recruitment, exposure, follow-up, and data collection	4-5
Participants	6	(*a*) Give the eligibility criteria, and the sources and methods of selection of participants. Describe methods of follow-up	4-5
		(*b*) For matched studies, give matching criteria and number of exposed and unexposed	n/a
Variables	7	Clearly define all outcomes, exposures, predictors, potential confounders, and effect modifiers. Give diagnostic criteria, if applicable	4-5
Data sources/ measurement	8*	For each variable of interest, give sources of data and details of methods of assessment (measurement). Describe comparability of assessment methods if there is more than one group	4-5
Bias	9	Describe any efforts to address potential sources of bias	10
Study size	10	Explain how the study size was arrived at	4-5
Quantitative variables	11	Explain how quantitative variables were handled in the analyses. If applicable, describe which groupings were chosen and why	5-9
Statistical methods	12	(*a*) Describe all statistical methods, including those used to control for confounding	5-9
		(*b*) Describe any methods used to examine subgroups and interactions	5-9
		(*c*) Explain how missing data were addressed	n/a
		(*d*) If applicable, explain how loss to follow-up was addressed	4
		(*e*) Describe any sensitivity analyses	n/a
**Results**			

For peer review only

Participants	13*	(a) Report numbers of individuals at each stage of study—eg numbers potentially eligible, examined for eligibility, confirmed eligible, included in the study, completing follow-up, and analysed	6
		(b) Give reasons for non-participation at each stage	6
		(c) Consider use of a flow diagram	n/a
Descriptive data	14*	(a) Give characteristics of study participants (eg demographic, clinical, social) and information on exposures and potential confounders	6-9
		(b) Indicate number of participants with missing data for each variable of interest	6-9
		(c) Summarise follow-up time (eg, average and total amount)	4
Outcome data	15*	Report numbers of outcome events or summary measures over time	n/a
Main results	16	(*a*) Give unadjusted estimates and, if applicable, confounder-adjusted estimates and their precision (eg, 95% confidence interval). Make clear which confounders were adjusted for and why they were included	n/a
		(*b*) Report category boundaries when continuous variables were categorized	n/a
		(*c*) If relevant, consider translating estimates of relative risk into absolute risk for a meaningful time period	n/a
Other analyses	17	Report other analyses done—eg analyses of subgroups and interactions, and sensitivity analyses	n/a
**Discussion**			
Key results	18	Summarise key results with reference to study objectives	6-9
**Limitations**			
Interpretation	20	Give a cautious overall interpretation of results considering objectives, limitations, multiplicity of analyses, results from similar studies, and other relevant evidence	6-11
Generalisability	21	Discuss the generalisability (external validity) of the study results	10-11
**Other information**			
Funding	22	Give the source of funding and the role of the funders for the present study and, if applicable, for the original study on which the present article is based	12

*Give information separately for cases and controls in case-control studies and, if applicable, for exposed and unexposed groups in cohort and cross-sectional studies.

Note: An Explanation and Elaboration article discusses each checklist item and gives methodological background and published examples of transparent reporting. The STROBE checklist is best used in conjunction with this article (freely available on the Web sites of PLoS Medicine at http://www.plosmedicine.org/, Annals of Internal Medicine at http://www.annals.org/, and Epidemiology at http://www.epidem.com/). Information on the STROBE Initiative is available at www.strobe-statement.org.

For peer review only
